# Numerical, phenotypic and functional differences in human dermal resident memory T cells between reticular dermal layers

**DOI:** 10.1002/ski2.457

**Published:** 2024-12-01

**Authors:** Takuya Sato, Riko Asakawa, Youichi Ogawa, Yuka Nagasaka, Manao Kinoshita, Shinji Shimada, Akira Momosawa, Tatsuyoshi Kawamura

**Affiliations:** ^1^ Department of Dermatology Faculty of Medicine University of Yamanashi Yamanashi Japan; ^2^ Department of Plastic Surgery Faculty of Medicine University of Yamanashi Yamanashi Japan

## Abstract

**Background:**

Resident memory T (T_RM_) cells and immunosuppressive Foxp3‐expressing regulatory T_RM_ (regT_RM_) cells are present in healthy, non‐inflamed and inflamed human skin. Both types of cells are found in both the epidermis and dermis with the dermis being much thicker than the epidermis. However, it is unclear if T_RM_ and regT_RM_ cells differ between reticular dermal layers in terms of number, function or characteristics.

**Methods:**

This study examined numerical, phenotypic and functional differences in T_RM_ and regT_RM_ cells between the upper and lower reticular dermis in healthy, non‐inflamed human skin using flow cytometry.

**Results:**

The phenotype and cytokine expressions of both types of cells were similar between reticular dermal layers. However, the cell count of both T_RM_ and regT_RM_ cells was significantly higher in the upper reticular dermis than in the lower reticular dermis.

**Conclusions:**

T_RM_ and regT_RM_ cells are phenotypically and functionally identical between reticular dermal layers. However, both types of cells are more abundant in the upper reticular dermis.

**What is already known?**

- Resident memory T (T_RM_) cells and immunosuppressive Foxp3‐expressing regulatory T_RM_ (regT_RM_) cells are present in human skin. Both types of cells are found in both the epidermis and dermis.

**What does this study add?**

- TRM cells and regT_RM_ cells are phenotypically and functionally identical between reticular dermal layers. However, both types of cells are more abundant in the upper reticular dermis.

## 1 INTRODUCTION

Recent advances have revealed that resident memory T (T_RM_) cells are present in the epidermis and dermis of healthy, non‐inflamed and inflamed human skin.^1^, ^2^ Moreover, immunosuppressive Foxp3‐expressing regulatory T_RM_ (regT_RM_) cells are also present in the epidermis and dermis.^3^, ^4^, ^5^ The composition of T_RM_ cells is different between the epidermis and dermis. CD8^+^ T_RM_ cells are significantly more abundant in the epidermis than in the dermis, whereas CD4^+^ T_RM_ cells are significantly more abundant in the dermis than in the epidermis.^2^ However, these results were derived from the cells of the epidermis and papillary/subpapillary dermis because the reticular dermis must be trimmed for efficient enzymatic separation of the epidermis from the underlying dermis. Given that the dermis is much thicker than the epidermis, it is likely that T_RM_ and regT_RM_ cells exhibit numerical, phenotypic or functional differences between dermal layers. Hence, this investigation identified numerical, phenotypic or functional differences in T_RM_ and regT_RM_ cells between the upper and lower reticular dermis using healthy non‐inflamed human skin.

## 2 MATERIALS AND METHODS

### 2.1 Ethical approval and sources of tissues

The Institutional Review Board of the University Hospital (University of Yamanashi, Yamanashi, Japan) approved the acquisition of human tissues and informed consent was obtained from all skin donors. Healthy non‐inflamed human samples were obtained from routinely discarded tissue following plastic surgery. Unless otherwise noted, healthy and non‐inflamed human skin from female breast was used in the experiments.

Thirteen skin samples were used in this study.

### 2.2 Tissue processing

RPMI 1640 (Invitrogen Life Technologies) containing 10% fetal bovine serum (Biowest) and Anti–Anti (1:100; Gibco) without additional exogenous cytokines was utilized as a culture medium. This study analyzed cells from the dermis recovered using the spontaneous migration method. At day 0, the skin was washed with sterile cold phosphate‐buffered saline (PBS) (Gibco) immediately after surgery. To obtain the lower dermis, the subcutaneous fat was thoroughly removed using a pair of scissors. Subsequently, the dermis that was approximately 1‐mm thick and near the subcutaneous fat was removed and used as the lower dermis. To avoid T_RM_ cells in the middle layer from contaminating the upper and lower dermis, this layer was trimmed. Subsequently, the approximately 1‐mm thick dermis that was near the middle layer was removed and used as the upper dermis. The epidermis, papillary dermis and subpapillary dermis were discarded. This means that the upper and lower dermis defined in this study refer to the upper and lower portions of the reticular dermal layer. To obtain emigrants, the lower and upper dermis pieces were floated separately in the culture medium for 2 days at 37°C. At day 2, these emigrants were analyzed.^2^

### 2.3 Flow cytometry

The following monoclonal antibodies (mAbs) were used in this study: anti‐CD3 (clone: HIT3a), anti‐CD4 (clone: OKT‐4), anti‐CD8a (clone: HIT8a and RPA‐T8), anti‐CD69 (clone: FN50), anti‐CD103 (clone: Ber‐ACT8), anti‐CD45 (clone: 2D1), anti‐IFN‐γ (clone: B27) and anti‐TNF‐α (clone: MAb‐11). These mAbs were purchased from BioLegend. Anti‐Foxp3 (clone: PCH101) was purchased from eBioscience™.

Cells were incubated with mAbs for 30 min at 4°C and then washed twice in staining buffer and examined using FACS Celesta (BD Biosciences). Dead cells were labelled by propidium iodide (Sigma‐Aldrich) or LIVE/DEAD™ Fixable Violet Dead Cell Stain Kit (Invitrogen™). Data were analyzed using the FlowJo software (FlowJo, LLC).

For Foxp3 staining, surface antigens were stained as described above, followed by cell fixation and permeabilization using eBioscience™ Foxp3/Transcription Factor Staining Buffer Set (Invitrogen™) according to the manufacturer's instructions. Cells were incubated with anti‐Foxp3 mAb for 30 min at 4°C and then washed twice in the buffer.

For cytokine expression, cells were cultured at a density of 1−2 × 10^5^ per 96‐well round bottom culture plate with 200 μL of culture medium supplemented with eBioscience Cell Stimulation Cocktail (500×) and eBioscience Protein Transport Inhibitor Cocktail (500×) from Invitrogen for 5 h at 37°C. Cultured cells were collected and surface antigens were stained as described above. Cells were fixed and permeabilized using Cytofix/Cytoperm reagents (BD Biosciences) for 20 min at 4°C, incubated with mAbs for cytokines for 30 min at 4°C and then washed twice in the buffer.

### 2.4 Methods for determining absolute cell counts

Cold PBS (Gibco) was placed in a 10‐cm dish and weighed. The upper and lower reticular dermis were collected separately in a 10‐cm dish and reweighed. The weight of the dermis was determined by subtracting it from the weight before the dermis was added. All cell counts of emigrants obtained after 2‐day culture were counted, including dead cells. The absolute cell number of live lymphocytes per gram (Figure 2f) was calculated by multiplying the total cell count by the % of the lymphocyte gate and % of the live cells determined by flow cytometry and dividing it by the initially measured weight. In addition, the absolute cell count per gram (Figures 2g and 3c) was calculated by multiplying the absolute cell count of live lymphocytes per gram (Figure 2f) by the % of the indicated cell types by flow cytometry.

### 2.5 Quantitative PCR

Total RNA was extracted from human dermis using QIAzol® Lysis Reagent (Qiagen) and RNeasy® Plus Universal Mini kit (Qiagen) as per the manufacturer's instructions. Reverse transcription was performed using ReverTra Ace® qPCR RT Kit (Toyobo) as per the manufacturer's instructions. mRNA levels were determined using commercially available primer/probe sets (TaqMan® Gene Expression Assay: Applied Biosystems) and the AB7500 real‐time polymerase chain reaction (PCR) system (Applied Biosystems). The amount of target gene mRNA obtained using real‐time PCR was normalized against the amount of housekeeping control gene (*ACTB*) mRNA. Human *TGF‐β1* primer was designed by Takara.

### 2.6 Statistical analysis

Comparisons were evaluated using Student's *t*‐test (two‐tailed) or one‐way ANOVA; ns means not significant, and *p < 0.05, **p < 0.01 and ***p < 0.001.

## 3 RESULTS

**FIGURE 1:**
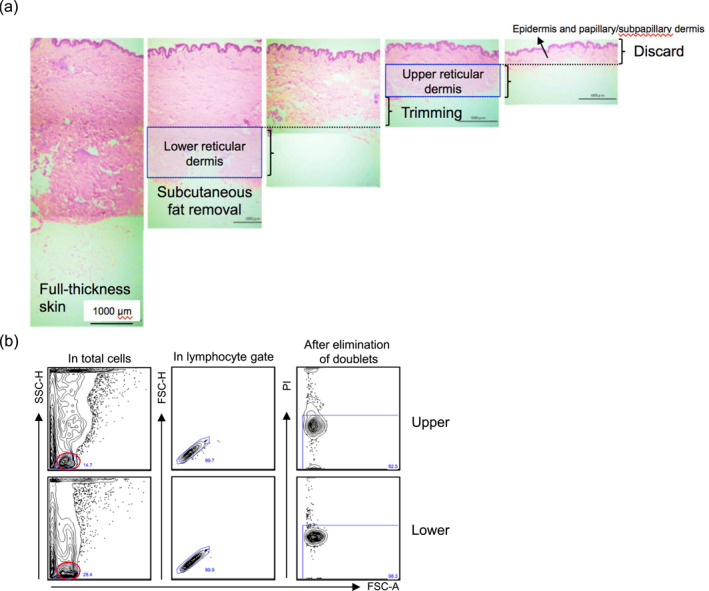
(a) Preparation of the upper and lower reticular dermis (haematoxylin‐eosin staining). The original magnification is ×100. (b) Gating strategy for flow cytometry. In total emigrants, the lymphocyte population indicated that a red circle was gated, followed by the elimination of doublets and dead cells. FSC, forward scatter; PI, propidium iodide; SSC, side scatter.

**FIGURE 2:**
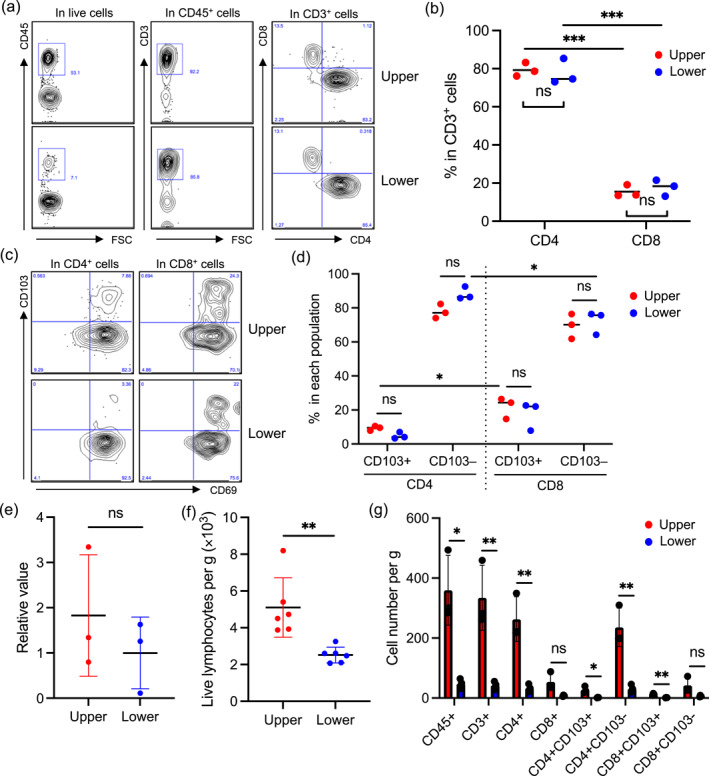
(a) Gating strategy for flow cytometry. CD45^+^ and CD3^+^ T cells were gated in live cells, followed by the segregation of CD4^+^ and CD8^+^ T cells. (b) A summary of the percentage of CD4^+^ and CD8^+^ T cells in CD3^+^ T cells (*n* = 3). (c) Representative CD69 and CD103 expression in CD4^+^ and CD8^+^ T cells. (d) A summary of the percentage of CD103^+^ and CD103^−^ T cells in CD4^+^ and CD8^+^ T cells (*n* = 3). (e) *TGF‐β1* mRNA expression in the upper and lower reticular dermis (*n* = 3). (f) A summary of absolute cell number per g of the upper and lower reticular dermis (*n* = 5). (g) A summary of absolute cell number per gram of indicated types of cells (*n* = 3). Comparisons between the upper and lower reticular dermis, and CD103^+^ and CD103^−^ T cells were evaluated using Student's *t*‐test (two‐tailed) and comparisons between CD4^+^ and CD8^+^ T cells were evaluated using one‐way ANOVA; ns means not significant, and *p < 0.05, **p < 0.01 and ***p < 0.001.

Upper and lower reticular dermis samples were obtained from healthy and non‐inflamed human skin immediately after surgery (Figure 1a). The spontaneous migration method was utilized to analyze dermal T_RM_ cells because enzymatic digestion of the dermis with collagenases strongly cleaves surface antigens, such as CD4, CD8, CD69 and CD103.^2^ Conversely, the spontaneous migration method recovers and/or upregulates antigens with T‐cell activation and loses ∼20% of T cells in the floating sheets. However, there was no big bias regarding CD103 expression between emigrants and the remaining T cells in the sheets.^2^ To obtain emigrants, lower and upper reticular dermis pieces were floated separately in the culture medium for 2 days at 37°C. Subsequently, the emigrants were subjected to flow cytometry. The lymphocyte population was gated in total emigrants, followed by the elimination of doublets and dead cells (Figure 1b). Subsequently, CD45^+^ cells were gated, followed by the gating CD3^+^ cells. CD45^+^CD3^+^ dermal T cells were segregated into CD4^+^ and CD8^+^ T cells (Figure 2a). The percentage of CD4^+^ and CD8^+^ T cells in CD3^+^ T cells was comparable between the upper and lower reticular dermis. However, the percentage of CD4^+^ T cells was significantly greater than that of CD8^+^ T cells in both reticular dermal layers (Figure 2b). Next, the expression of CD69 and CD103 was examined in the CD4^+^ and CD8^+^ T cells. Consistent with previous reports,^1^, ^2^ both the CD4^+^ and CD8^+^ T cells in both reticular dermal layers almost entirely expressed CD69, a T_RM_ marker (Figure 2c). The percentage of CD103^−^ cells in CD4^+^ and CD8^+^ T_RM_ cells of both reticular dermal layers was significantly greater than that of CD103^+^ cells. In the four groups (CD4^+^ or CD8^+^, and CD103^+^ or CD103^−^), no difference was observed between the reticular dermal layers (Figure 2d). However, minor but significant differences were observed: CD103^+^ cells were greater in CD8^+^ T_RM_ cells than in CD4^+^ T_RM_ cells in the upper reticular dermis. Moreover, CD103^−^ cells were greater in CD4^+^ T_RM_ cells than in CD8^+^ T_RM_ cells in the lower reticular dermis (Figure 2d). Rather unexpectedly, the percentage of CD103^+^ cells was not altered between the reticular dermal layers. One possible explanation was that *TGF‐β1* mRNA expression was comparable between reticular dermal layers, which is indispensable for the induction of CD103 expression (Figure 2e). Collectively, the phenotypic difference in T_RM_ cells was not evident between the reticular dermal layers. However, the absolute cell number per gram of the upper and lower reticular dermis was significantly greater in the upper reticular dermis than in the lower reticular dermis (Figure 2f). Accordingly, the upperreticular dermis contained significantly greater CD45^+^, CD3^+^, CD4^+^CD103^+^, CD4^+^CD103^−^, and CD8^+^CD103^+^ cells than the lower reticular dermis (Figure 2g).

**FIGURE 3:**
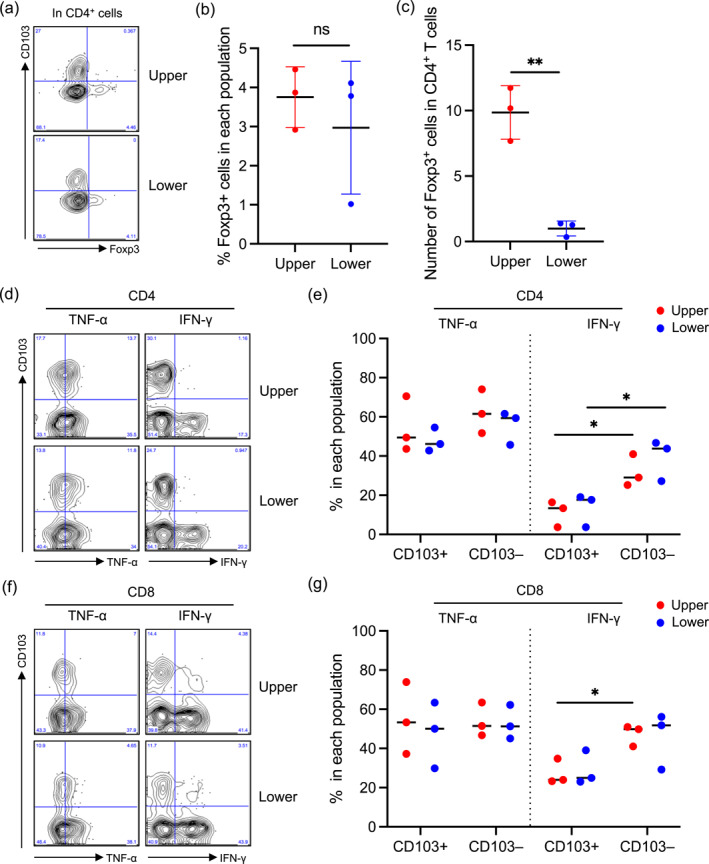
(a) Representative forkhead box p3 (Foxp3) and CD103 expression in CD4^+^ T cells. (b) A summary of the percentage of Foxp3^+^ cells in CD4^+^ T cells (*n* = 3). (c) A summary of absolute Foxp3^+^ cell number per g of the upper and lower reticular dermis (*n* = 3). (d and f) Representative TNF‐α and IFN‐γ expression in CD4^+^ (d) and CD8^+^ (f) T cells. (e and g) A summary of the percentage of TNF‐α^+^ and IFN‐γ^+^ cells in CD103^+^ and CD103^−^ T cells of CD4^+^ (d) and CD8^+^ (f) T cells. Comparisons between the upper and lower reticular dermis, and CD103^+^ and CD103^−^ T cells were evaluated using Student's *t*‐test (two‐tailed); ns means not significant, and *p < 0.05, **p < 0.01, and ***p < 0.001. IFN‐γ, interferon‐gamma; TNF‐α, tumour necrosis factor‐alpha.

The percentage of Foxp3^+^ in CD4^+^ T_RM_ cells was consistently similar between the reticular dermal layers (Figure 3a,b). However, the absolute regT_RM_ cell number was greater in the upper reticular dermis than in the lower reticular dermis (Figure 3c) because the absolute CD4^+^ T_RM_ cell number was greater in the upper reticular dermis (Figure 2g). Functional differences in CD4^+^ and CD8^+^ T_RM_ cells between reticular dermal layers were examined by determining the expression of tumour necrosis factor‐alpha (TNF‐α) and interferon‐gamma (IFN‐γ). TNF‐α expression by CD4^+^ and CD8^+^ T_RM_ cells was similar between reticular dermal layers irrespective of CD103 expression (Figure 3d–g). Moreover, IFN‐γ expression by CD4^+^ and CD8^+^ T_RM_ cells was similar between reticular dermal layers (Figure 3d–g). However, IFN‐γ expression by CD4^+^ T_RM_ cells was greater in CD103^−^ cells than in CD103^+^ cells in both the upper and lower reticular dermis (Figure 3d,e). In addition, IFN‐γ expression by CD8^+^ T_RM_ cells was greater in CD103^−^ cells than in CD103^+^ cells in the upper reticular dermis (Figure 3f,g).

## 4 DISCUSSION

This study confirmed that phenotype and cytokine expression of T_RM_ and regT_RM_ cells were similar between the upper and lower reticular dermis. However, the cell number of both types of cells is significantly greater in the upper reticular dermis than in the lower reticular dermis. Predominant cell distribution in the upper dermis is also found in mast cells and innate lymphoid cells.^6^, ^7^ The numerical predominance of these immune cells in the upper dermis is useful when preparing for trans‐epidermal exposure to external antigens. However, its immunological mechanisms are unknown. A possible explanation is that dermal layer‐specific factors, such as the density of nerves or vessels, exposure to external antigens and ultraviolet irradiation, and frequency of minor trauma, influence the regulation of the abundance of immune cells in the upper dermis. Specifically, CD8^+^ and CD4^+^ T_RM_ cells are predominant T cells in the epidermis and dermis, respectively.^2^ However, T_RM_ cell composition is similar between reticular dermal layers. In addition, Foxp3 is selectively expressed in CD103^−^ cells of ‘epidermal’ regT_RM_ cells because it is highly demethylated.^5^ In line with this finding, Foxp3 is also selectively expressed in CD103^−^ cells of ‘reticular dermal’ regT_RM_ cells. Finally, various cytokines are preferentially produced by CD103^+^ cells of ‘epidermal’ T_RM_ cells.^1^, ^5^ However, at least TNF‐α and IFN‐γ are preferentially produced by CD103^−^ cells of ‘reticular dermal’ T_RM_ cells. Studies regarding human skin T_RM_ cell biology are early in their development. Therefore, the accumulation of basic evidence may help in the scientific advancement of this field.

## 5 CONCLUSION

T_RM_ and regT_RM_ cells are phenotypically and functionally identical between reticular dermal layers. However, both types of cells are more abundant in the upper reticular dermis.

## CONFLICT OF INTEREST STATEMENT

None to declare.

## AUTHOR CONTRIBUTIONS

**Takuya Sato**: Data curation (lead); formal analysis (lead); investigation (lead); project administration (lead). **Riko Asakawa**: Data curation (lead); formal analysis (lead); investigation (lead); project administration (lead). **Youichi Ogawa**: Conceptualization (lead); funding acquisition (lead); project administration (lead); validation (lead); visualization (lead); writing—original draft (lead). **Yuka Nagasaka**: Investigation (supporting); methodology (supporting); project administration (supporting); resources (equal); writing—review & editing (supporting). **Manao Kinoshita**: Methodology (supporting); project administration (supporting); software (supporting); writing—review & editing (supporting). **Shinji Shimada**: Project administration (supporting); supervision (supporting); validation (supporting); writing—review & editing (supporting). **Akira Momosawa**: Conceptualization (supporting); project administration (supporting); resources (lead); supervision (supporting); writing—review & editing (supporting). **Tatsuyoshi Kawamura**: Conceptualization (supporting); project administration (supporting); supervision (supporting); writing—review & editing (supporting).

## ETHICS STATEMENT

The Institutional Review Board of the University Hospital (University of Yamanashi, Yamanashi, Japan) approved the acquisition of human tissues, and informed consent was obtained from all skin donors.

## PATIENT CONSENT

Written patient consent for publication was obtained.

## Data Availability

The data underlying this article will be shared on reasonable request to the corresponding author.
